# Lifetime employment histories and their relationship with 10-year health trajectories in later life: evidence from England

**DOI:** 10.1093/eurpub/ckaa008

**Published:** 2020-02-24

**Authors:** Giorgio Di Gessa, Laurie Corna, Debora Price, Karen Glaser

**Affiliations:** c1 Research Department of Epidemiology and Public Health, University College London, 1-19 Torrington Place, London, UK; c2 Department of Business Economics, Health and Social Care, Centre of Competence on Ageing, University of Applied Sciences and Arts of Southern Switzerland, Manno, Switzerland; c3 School of Social Sciences, Manchester Institute for Collaborative Research on Ageing, University of Manchester, Manchester, UK; c4 Department of Global Health and Social Medicine, Institute of Gerontology, King’s College London, London, UK

## Abstract

**Background:**

Employment histories influence health. However, most studies have so far investigated cross-sectional associations between employment histories and health, failing to recognize health as a dynamic process in later life.

**Methods:**

We use Waves 3–8 of the English Longitudinal Study of Ageing, including retrospective information on respondents’ employment activities. We used dynamic hamming distances to summarize lifetime employment histories up to state pension age (64 for men and 59 for women). Multilevel growth curve models were then used to estimate the influence of lifetime employment histories on later life health trajectories over a 10-year period using quality of life (QoL), somatic health, and depression.

**Results:**

Net of selection effect and a host of contemporaneous material and social resources, men who exited early started off with poorer health than those with continuous attachment to the labour market but had a very similar health profile by the end of the 10-year period considered. Among women, better somatic health and higher QoL were observed among those who had employment breaks for family care, and this health advantage was maintained over time. Lifetime employment histories are not related to depression for either men or women.

**Conclusion:**

Overall, differences in health by employment histories level off only among men who left earlier and those continuously employed. Flexible arrangements for men in poor health who benefit from leaving the labour market early and supporting women who wish to take breaks for family care may help reduce health inequalities in later life.

## Introduction

Research has consistently shown a positive relationship between paid work and health: at any given point, employed individuals report better health than the unemployed individuals or those out of the labour force^1^ but in this, mainly cross-sectional work, neither work nor health are considered as a dynamic processes varying over time. More recently, the cumulative effects of labour market experiences over time on health at older ages have been considered, showing that, e.g. those who had more unstable and weaker lifetime labour market attachment tend to report poorer health at one or two time points in mid and later life^2–14^ relative to those with stronger ties. Yet, employment histories may continue to have an enduring effect on health even past state pension age (SPA). This is likely to be driven by a number of mechanisms, including selection effects and material and social resources that accumulate over time. Little is known about the longer-term health trajectories associated with various employment histories. This is an important oversight as understanding whether health differentials associated with particular patterns are maintained well past SPA, or whether they level off over time, is instructive for informing employment and retirement policies.

Using data from the English Longitudinal Study of Ageing (ELSA), we assess how lifetime labour market experiences are associated with health trajectories over a 10-year period among men and women who are at or beyond the SPA, net of selection effects and a host of contemporaneous material and social resources.

### Background

Our understanding of how lifetime labour market experiences are related to mid- and later-life health has grown considerably since the early 2000s. For example, studies show that men with continuous labour market attachment across the ‘working years’ report more favourable health than their counterparts with relatively weak ties.^5^^,^^10^ Among women, those who combine shorter periods of family care with strong labour market attachment are healthier in their mid-50s than those who spend longer periods of time out of the labour market,^4^ and their risk of mortality and disability at older ages is also lower compared with their counterparts with histories of continuous paid work or absence from the labour market,^9^ but this may differ in different national contexts.^15^ Women who interrupt paid work with breaks for family care, followed by employment up to SPA, also report the highest quality of life (QoL) in later life.^5^ For women, occupying multiple roles has been shown to be health enhancing^16^^,^^17^ but temporarily interrupting employment to care for young families may confer social benefits, such as the avoidance of role burden, which could have an enduring effect on health. Both men and women with stronger ties to the labour market may be more likely to have accumulated material (e.g. wealth, pension entitlements, home ownership) and social resources (e.g. health-enhancing connections) which favour better health post-SPA.^18^

Although previous studies suggest that employment trajectories are differentially associated with health, prior research has been limited in several ways. Most notably, studies often rely on health data collected at one time point in mid life^4^^,^^6^^,^^7^ or later life.^4^^,^^6–9^^,^^11–14^ This precludes the possibility of understanding the longer-term influence of work experiences on dynamics in health as individuals age, an important consideration for informing employment and retirement policies. Sustained differences in health trajectories by work histories in the years post-SPA, net of selection effects and material and social resources, suggest that employment experiences leave their mark on individuals above and beyond proposed mechanisms.

Moreover, men are frequently excluded from analyses as their employment histories are less constrained by family responsibilities.^4^^,^^8^^,^^9^^,^^11–14^ When they are included, summaries of employment histories use the same categories for both sexes, overlooking key, and potentially consequential, differences in the lifetime employment experiences of men and women.^4–7^ Also, few studies have adjusted for potential selection effects (i.e. early-life health and socio-economic factors) thought to influence both employment opportunities and histories, as well as subsequent health.^8^^,^^9^^,^^11–14^

Our study investigates the associations between lifetime employment histories up to SPA and 10-year trajectories of three measures of health (QoL, somatic health, and depression) among men and women post-SPA in England, taking account of selection effects and contemporaneous material and social factors.

## Methods

### Study population

We used data from the third (2006/07) to the eighth wave (2016/17) of ELSA, a multidisciplinary longitudinal survey of individuals aged 50 and over living in England (http://www.elsa-project.ac.uk/). Our analytical sample included respondents who participated in Wave 3 (when retrospective data were collected) and who had reached SPA by that wave (65 for men, 60 for women^19^) This sample was further restricted to men aged 65–74 and women aged 60–69 at Wave 3 to avoid having too many losses to follow-up and deaths over the 10-year period under study. After excluding respondents who had died (*n* = 128, 5.9%) and who only had data at baseline (*n* = 89, 4.4%), the final sample consisted of 794 men and 1140 women, of which 68% (*N* = 1310) were present in all Waves under study.

### Measures

#### Outcomes

Our health outcomes are QoL, somatic health, and depression, all associated with increased mortality.^20–23^*Subjective quality of life (QoL)* was evaluated using the CASP-19 scale, a validated measure specifically designed for individuals in later life,^24^ with values ranging from 0 to 57 and higher scores indicating greater QoL. As a measure of *somatic health*, we derived a single latent health index as proposed by Ploubidis and Grundy.^25^ This index, which combines both self-reported health (such as self-rated health and limitations with activities of daily living) and observer-measured health information (walking speed), is less subject to measurement error than separate health indicators and has greater repeatability and reliability.^25^ Finally, *symptoms of depression* were measured using an abbreviated eight-item version of the validated Centre for Epidemiologic Studies Depression (CES-D) Scale. Those who reported three or more symptoms were classified as having depressive symptoms.^26^

#### Employment histories

The employment history data in wave 3 were used to derive individual histories of labour market involvement between the ages of 16 and 64 years for men, and 16 and 59 years for women. Individual economic activity was coded at each age, distinguishing between full-time employment, part-time employment (≤30 h/week) and other activities (unemployment, provision of family care, incapacity, education, and retirement). The patterns of economic activity experienced by men and women over their lives were classified using sequence analysis, a group of research methods which study whole sequences and investigate the timing, order, and duration of events, providing a nuanced account of lifetime experiences.^27^ In particular, in line with the focus on the timing of employment events, we used dynamic hamming distances to group whole sequences of employment experiences. This method relies exclusively on substitutions to preserve the timing of role occupancies. The algorithm allocates substitution costs according to how often transitions happen in the data between two consecutive points.^27^ We used an ‘ideal type’ comparison method which compares all observed sequences against a set of ideal type trajectories,^28^ as described fully in Corna *et al.*^29^ We considered five ideal employment histories for men (employed full-time throughout; not employed throughout; full-time up to 59; early exit at 49; and start of paid work at 23 and exit at 60) and seven for women (employed full-time throughout; employed mostly part-time throughout; not employed throughout; early exit at 48; with a short career break between 26 and 30 followed by part-time employment; with a long career break between 26 and 41 followed by part-time employment; and with a medium career break between 26 and 34 followed by full-time employment). However, given the small number of cases in some categories, we combined selected groups that included respondents with conceptually similar employment histories (see table 1 for details; see also Supplementary figures S1 and S2). It is important to note that each individual is assigned to their closest ‘ideal type’. Robustness checks performed using traditional optimal matching^30^ yielded very similar classifications of individuals’ employment histories.

**Table 1:** Per cent (and *N*) distribution of lifetime employment histories by gender

		All sample
MEN	Continuous work mostly FT up to SPA	46.5 (369)
	Weak labour market attachment	
	Mostly non-employed throughout	3.5 (28)
	Full-time very early exit (at about age 49)	11.6 (92)
	Full-time early exit (at about age 60)	28.1 (223)
	Late start at about age 23, early exit (at about 60)	10.3 (82)
		794
	Continuous work FT or PT up to SPA	
WOMEN	Mostly part-time throughout to SPA	5.4 (61)
	Mostly full-time throughout to SPA	27.7 (316)
	Weak labour market attachment	
	Mostly non-employed throughout/family carer	16.1 (183)
	Early exit (at about age 48)	6.6 (75)
	Long Break (about ages 26–41) to PT up to SPA	11.9 (136)
	Medium Break (about 26–34) to FT up to SPA	19.0 (217)
	Short Break (about 26–30) to PT up to SPA	13.3 (152)
		1140

*Notes:* It is important to acknowledge that individuals in each of these categories are mostly/always employed or non-employed around the ages indicated because cases are matched to their closest model sequence. FT and PT stand for full time and part time, respectively. SPA was 60 for women and 65 for men.

*Source:* ELSA Life History, 2006/2007.

#### Covariates

We controlled for a wide range of material and social resources in later life that are known to be associated with either or both health and lifetime employment histories. We also included socio-economic and health circumstances in childhood, and health in adulthood to address the issue of selection into particular employment histories. For the former, we combined several indicators of early life socio-economic circumstances (SEC) and health in childhood (up to age 16) using latent class analysis (LCA), as this allowed us to classify respondents into homogenous subgroups (see Supplementary figure S3 for a full list of variables). As controls for health selection, we considered the number of periods of ill health or disability lasting more than a year, and whether respondents ever left a job because of ill-health. Moreover, we controlled for age (centred at SPA); educational qualifications; social class (managerial and professional; intermediate; and routine and manual occupations); number of children ever born; and experiences of marital breakup through divorce or widowhood. Time-varying covariates were wave and wave squared, total net non-housing wealth and income (created by the Institute for Fiscal Studies^31^) housing tenure; partnership status (living with partner vs. not). We also considered the number of close relationships; the extent of positive support; and the frequency of contacts with family and friends. Finally, we controlled for smoking (non-smoker vs. current smoker) and vigorous physical activity (at least weekly vs. less often).

### Statistical analysis

Given repeated measures of health outcomes within individuals, multilevel modelling was used to estimate growth curve models of health by lifetime employment histories (allowing for random intercepts and slopes) using a maximum likelihood algorithm. In light of significant gender differences in employment histories, we carried out analyses separately for men and women. Given our interest in whether our sample, a group of people born in the same decade, differentially experienced changes in health based on their employment histories, we opted for period trajectories over age trajectories, modelling health as a function of time using a quadratic function (wave and wave 2) to allow for non-linear changes. We first adjusted for selection (Model I, early SEC and health; health in adulthood); followed by two models that accounted for contemporaneous material (Model II, socio-economic characteristics and health behaviours) and social resources (Model III, marital and fertility histories as well as social contacts and support). Multiple imputations (MI) were used for dealing with missing values in the main exposure and other covariates. These were carried out in two stages: first, work states were imputed by Anonymous creating 20 imputed data sets. Then we augmented them separately by gender with imputation of missing information on the covariates and outcomes using MI by chained equations. The results of these analyses were then combined using Rubin’s rules.^32^ Latent summaries and LCA were estimated using Mplus7; all other analyses were performed in Stata 15.

## Results

### Measurement models

We considered a unidimensional model to summarize measures of somatic health. As shown in the Supplementary table S1, the model fits the data well. The latent variable offers a continuous measure, where positive high scores indicate good somatic health.

We classified respondents’ childhood health and SEC into four homogenous subgroups, as these provided the best model fit. As shown in Supplementary figure S3, the four groups represent the combination of lower/higher SEC and good/poor health in childhood.

### Descriptive statistics

Table 2 presents the baseline sample characteristics by lifetime employment histories. Overall, men who were late starters with an early exit, and women who had family breaks, tended to report better health at baseline than those with continuous attachment to the labour market. Men and women with weak attachment to the labour market had poorer health, and also tended to be in routine and manual occupations, to smoke, be physically inactive, and to have experienced poor health in adulthood, while also having a high number of close relationships and frequent contacts with friends and family.

**Table 2:** Baseline sample characteristics (mean/percentages) by gender-specific categories of lifetime employment histories

	Men					Women					
	Continuous work	Weak attachment	Exit at 60	Late start	*P*-value	Continuous work	Weak attachment	Long break	Medium break	Short break	*P*-value
Quality of life	43.58	41.10	41.87	45.56	<0.001	43.49	40.88	43.89	43.45	44.37	<0.001
Somatic health	1.01	−1.79	−0.39	1.98	<0.001	0.08	−1.88	1.11	1.13	1.09	<0.001
3+ Depressive symptoms	11.9	20.0	17.5	9.8	0.046	21.2	29.1	13.9	15.7	15.1	<0.001
Education											
Some education	74.1	60.5	71.4	100	<0.001	79.0	58.7	77.0	68.1	76.6	<0.001
No educational qualification	25.8	39.5	28.6	0		21.0	41.3	23.0	31.9	23.4	
Social class											
Managerial and professional	26.8	27.9	41.3	76.2	<0.001	36.8	17.0	21.7	17.2	40.4	<0.001
Intermediate	30.0	16.3	14.6	15.5		29.8	28.4	32.6	33.1	25.3
Routine and manual	43.2	55.8	44.1	8.3		33.4	54.6	45.7	49.7	34.2
Age (mean)	69.1	68.1	68.9	67.9	<0.001	63.2	63.6	62.9	63.6	63.1	0.086
Home tenure											
Own outright	77.6	62.3	77.8	84.5	<0.001	69.4	66.5	73.2	75.5	66.1	<0.001
Own with mortgage	11.6	10.0	6.9	13.1		19.7	12.3	18.1	16.1	22.3
Non-owners	10.8	27.7	15.3	2.4		10.9	21.2	8.7	8.4	11.6
Smoker	11.1	21.7	13.5	4.9	0.003	15.9	16.3	8.8	10.1	13.2	0.087
No vigorous physical exercise	61.5	75.0	68.6	63.4	0.037	70.8	75.6	66.9	59.9	68.4	0.006
In a partnership	67.7	55.4	63.3	66.7	0.083	40.8	59.4	61.9	76.4	56.6	<0.001
Ever divorced/widowed	4.1	9.2	4.0	5.9	0.106	15.9	10.5	12.5	8.6	19.4	0.015
Number of children											
No children	11.8	20.8	14.9	13.1	0.002	27.9	9.2	5.0	7.6	2.7	<0.001
1	13.9	15.4	21.8	4.7		20.1	15.1	21.6	16.6	15.5
2	44.5	33.1	37.9	47.6		30.5	37.3	36.7	47.1	49.6
3 +	29.8	30.7	25.4	34.5		21.5	38.4	36.7	28.7	32.3
Social Relationships and Support											
Close relationships	7.86	10.76	8.07	7.43	0.005	8.22	9.13	8.40	7.90	8.63	0.571
Contacts with family	5.62	5.59	5.37	4.57	0.014	5.54	6.43	6.37	6.33	6.48	<0.001
Contacts with friends	3.52	3.98	3.54	3.50	0.005	3.79	3.76	3.79	3.73	3.61	0.689
Positive support by family	22.22	22.67	21.61	21.47	0.617	22.2	24.0	24.7	24.4	24.7	<0.001
Positive support by friends	12.17	12.03	11.73	11.77	0.447	13.5	12.7	12.9	13.1	12.7	0.013
Ever left employment because of ill health	12.1	40.0	27.0	14.3	<0.001	22.2	28.0	17.3	14.0	19.9	0.007
Two or more periods of ill health in adulthood	9.0	24.6	16.9	16.7	<0.001	16.3	21.0	13.7	8.9	12.8	0.010
Childhood characteristics											
Lower socio-economic circumstances, good health	36.0	29.3	29.4	14.3	<0.001	21.7	23.3	18.0	26.8	23.5	0.429
Lower socio-economic circumstances, poor health	5.9	11.5	8.5	3.6		5.2	7.8	7.9	2.6	7.1
Higher socio-economic circumstances, good health	48.3	48.5	45.6	58.3		56.9	55.7	60.4	59.9	55.3
Higher socio-economic circumstances, poor health	9.8	10.8	16.5	23.8		16.3	13.3	13.7	10.8	14.2
Respondents (*N*)	369	120	223	82		377	258	136	217	152	

*Note: P*-values refer to the relevant statistical tests (i.e. *t*-test, ANOVA, or chi-square tests).

*Source:* ELSA Life History and Wave 3, 2006/07. Own calculations; unweighted data.

### Multilevel models of health by employment histories

Tables 3 and 4 present the main effects for the fully adjusted relationship (Model III) between employment histories and health for men and women, respectively. Although we focus on the fully adjusted model, it is worth noting that controlling for contemporaneous material and social resources did not dramatically change the associations observed in Model I. One exception is the attenuation of the higher QoL among men who exited early at 60 but were late starters once socio-economic characteristics and health behaviours were included. Full details of all models are presented in Supplementary tables S2 and S3.

**Table 3:** Multilevel fully adjusted models of three measures of health by gender-specific categories of lifetime employment histories, beta coefficients, and odd ratios (and 95% CIs)—results for MEN (*N* = 794)

	Quality of Life	Somatic Health	Depressed (3+ CESD)
Continuous work	Ref.	Ref.	Ref.
Weak attachment	-1.789 (-3.36; -0.22)	-1.055 (-1.99; -0.13)	1.08 (0.49; 2.40)
Early exit at 60	-0.781 (-2.01; 0.44)	-0.477 (-1.21; 0.26)	1.19 (0.62; 2.29)
Late start, early exit at 60	0.663 (-1.14; 2.47)	0.365 (-0.73; 1.46)	0.83 (0.28; 2.39)
Age	-0.174 (-0.34; -0.01)	-0.202 (-0.30; -0.10)	0.99 (0.91; 1.07)
Wave	-0.565 (-0.91; 0.21)	-0.319 (-0.50; -0.14)	0.88 (0.68; 1.14)
Wave squared	-0.038 (-0.10; 0.02)	-0.025 (-0.06; 0.01)	1.05 (1.00; 1.10)
Wave*Labour histories			
Weak attachment	0.289 (-0.08; 0.66)	0.197 (0.00; 0.39)	1.02 (0.84; 1.25)
Early exit at 60	0.253 (-0.03; 0.54)	0.072 (-0.09; 0.23)	0.88 (0.74; 1.04)
Late start, early exit at 60	0.415 (0.01; 0.83)	-0.036 (-0.26; 0.19)	1.00 (0.76; 1.31)

*Sources:* ELSA Waves 3–8 and ELSA Life History. The full models control for social class, home tenure, smoking, physical exercise, partnership status, wealth, income, marital disruption, number of children, social contacts and support, episodes of ill health, and childhood SEC and health. All detailed models can be accessed online (see Supplementary table S2). Own calculations.

**Table 4:** Multilevel fully adjusted models of three measures of health by gender-specific categories of lifetime employment histories, beta coefficients and odd ratios (and 95% CIs)—results for WOMEN (*N* = 1140)

	Quality of Life	Somatic Health	Depressed (3+ CESD)
Continuous work	Ref.	Ref.	Ref.
Weak attachment	-0.621 (-1.89; 0.65)	-0.710 (-1.45; 0.03)	1.22 (0.75; 1.98)
Long break	0.689 (-0.85; 2.27)	0.583 (-0.32; 1.48)	0.77 (0.41; 1.43)
Medium break	1.329 (0.01; 2.64)	0.896 (0.12; 1.67)	0.81 (0.48; 1.38)
Short break	0.341 (-1.15; 1.84)	0.586 (-0.28; 1.46)	0.66 (0.35; 1.22)
Age	-0.132 (-0.26; -0.00)	-0.131 (-0.21; -0.05)	1.02 (0.97; 1.07)
Wave	-0.245 (-0.53; 0.03)	-0.042 (-0.20; 0.11)	1.08 (0.91; 1.29)
Wave squared	-0.016 (-0.06; 0.03)	-0.032 (-0.06; -0.01)	0.99 (0.96; 1.02)
Wave*Labour histories			
Weak attachment	0.123 (-0.13; 0.38)	0.038 (-0.10; 0.18)	0.93 (0.82; 1.06)
Long break	-0.048 (-0.35; 0.26)	-0.088 (-0.26; 0.08)	0.97 (0.83; 1.14)
Medium break	-0.047 (-0.31; 0.21)	-0.112 (-0.26; 0.04)	0.92 (0.80; 1.06)
Short break	0.084 (-0.22; 0.38)	-0.004 (-0.17; 0.16)	0.96 (0.82; 1.13)

*Sources:* ELSA Waves 3–8 and ELSA Life History. The full models control for education, social class, home tenure, smoking, physical exercise, partnership status, wealth, income, marital disruption, number of children, social contacts and support, episodes of ill health, and childhood SEC and health. All detailed models can be accessed online (see Supplementary table S3). Own calculations.

In the fully adjusted model, only men with weak attachment to the labour market had significantly lower QoL (β = −1.789) and somatic health (β = −1.055) at baseline than those with continuous employment. Over time, compared with those with continuous labour market attachment, men with an early exit from the labour market at age 60 had a lower starting point but a slower decline in QoL and somatic health (marginally significant at 10%), while men who exited early at 60 but were late starters reported a slighter higher QoL and a significantly slower rate of decline. No differences by employment histories in depressive symptoms were noted for either the initial probability or rate of change in the fully adjusted model.

For women, after full adjustment, only those with medium breaks reported significantly higher QoL (β = 1.329) and somatic health (β = 0.896) in comparison to those with continuous employment histories. Like men, no differences by employment histories were observed for depression in the final model. Finally, the decline in health for all three outcomes was not significantly different by employment histories.

### Health trajectories by employment histories

Fully adjusted conditional trajectories of health for men and women are shown in Supplementary figure S4. Men who were late starters with an early exit had the best health on all measures and showed the slowest decline in QoL. Men with continuous attachment to the labour market started off with better health than those who exited early but were projected to have a very similar health profile by the end of the 10-year period considered. Trajectories for women suggest that those who had family breaks from paid work maintain better health than those with continuous employment. Both men and women with weak attachment to the labour market report the poorest health over time and show no signs of recovery.

## Discussion

Our findings show that men with weak labour market attachment, and those who exited early, were more likely to report poorer somatic health and QoL than those with continuous employment. However, at the end of the 10-year period considered, those who exited early had similar health outcomes to those who had continuous attachment to the labour market until SPA. This suggests that for men in poor health, leaving employment before SPA may slow down further declines in somatic health and QoL at older ages. This finding is consistent with other studies looking at frailty,^10^ self-rated health,^33^ and mental and physical health.^34^ Moreover, men aged 65–74 at baseline who were late starters with an early exit had better somatic health and a significantly slower decline of QoL than men with continuous labour market attachment up to SPA. This is not unexpected as these men were in the most socio-economically advantaged group (see table 2), and likely to have had better access to health services and material resources relevant for health^35^ throughout their lives, as well as the opportunity to retire earlier through choice.^36^^,^^37^

Women at the two extremes of labour market attachment (continuous employment; weak attachment) had relatively poorer somatic health and QoL at baseline compared with those who took (mostly medium-length) breaks from paid work for family care. Women who had taken breaks from paid work also reported higher QoL and somatic health over the 10-year follow-up period compared with women who had been continuously employed until SPA (see table 3 and Supplementary figure S4), even when selection effects, material and social resources were taken into account.

To our knowledge, this is the first paper to consider the relationship between lifetime labour market histories and trajectories of physical and psychological health over an extended period of time (10 years) among adults at or beyond the SPA at baseline. Our findings underscore the importance of employment histories for health and well-being in later life: even after accounting for potential selection effects, and numerous material and social resources, there is an enduring effect of employment histories on health trajectories in the years post-SPA. However, our contribution should be considered in light of several limitations. First, all measurements except for walking speed are self-reported and may have introduced bias. Recall bias in the retrospective accounting of work histories is also possible, although research demonstrates reliability and validity of retrospective data when life calendars are used, as was the case with ELSA.^38^ Second, our sample was small, and therefore, the analysis may have lacked power to detect significant relationships. For example, the association between medium-length breaks from paid work and later life health trajectories may have been significant because of the larger relative size of this group compared with that of women with short breaks. Third, we did not have detailed information on work-related characteristics; yet we know that physically demanding jobs, low control, and stressful psychosocial environments all contribute to health at older ages.^39^ Finally, while our research design does not enable us to entirely account for potential endogeneity in the employment–health relationship, we mitigate this possibility by looking at health trajectories, which are less prone to issues of reverse causality. Relatedly, future research might also confirm the associations found here among larger age ranges, modelling trajectories by age instead of time (wave).

Despite these limitations, our findings suggest that health disparities by labour market histories observed at SPA are then maintained with age over time, particularly among women with those having had breaks from paid work reporting better health throughout. Evidence for a levelling-off effect across labour market categories is only observed between men with continuous employment and those who exited the labour market earlier than SPA. A logical next step in this area of research would be systematic testing of potential explanatory mechanisms.

These results have important implications for policy. First, given the potential for early retirement to slow subsequent declines in health and well-being for those in poorer health, opportunities for earlier exits in such cases may be warranted. However, the financial well-being of individuals in these circumstances needs to be considered given the long-established link between poverty and health,^40^ since pension accumulation is strongly associated with paid work. Second, policies that facilitate time away from the labour market for women with caring responsibilities (beyond the traditional period of maternity leave) may prove important for maintaining women’s health in later life.

## Supplementary Material

ckaa008_Supplementary_Data
