# Supplementary material for: Lifetime employment histories and their relationship with 10-year health trajectories in later life: evidence from England
Source: Eur J Public Health. 2020 Feb 24;30(4):793–9. doi: 10.1093/eurpub/ckaa008 (PMC7445040; doi:10.1093/eurpub/ckaa008)
Supplement: ckaa008_Supplementary_Data [file ckaa008_supplementary_data.pdf]

Supplementary Table S1. Standardised factor loadings of the health indicators on the somatic health measurement model and descriptive criteria of model fit, at both baseline (Wave 3) and at the final follow-up (wave 8).

Health indicators	Somatic health at Wave 3	Somatic health at Wave 8
Walking speed	0.58***	0.56***
Self-rated health	0.74***	0.75***
Severe long standing illness	0.80***	0.79***
IADL limitations	0.88***	0.89***
ADL limitations	0.87***	0.86***
Diagnosed condition	0.48***	0.43***
Mobility limitations	0.92***	0.91***
**Criteria of Model Fit**		
Comparative Fit Index	0.977	0.978
Tucker Lewis Index	0.984	0.980
Root Mean Square Error of Approximation (RMSEA)	0.059	0.051

Notes: All indicators were recoded such that high values represent good health. Values of the Comparative Fit Index and of the Tucker Lewis Index greater than 0.95 indicate good fit; values of the Root Mean Square Error of Approximation (RMSEA) less than 0.06 indicate good fit. ***: significant at $p<0.001$

Supplementary Figure S1. Categories of employment histories – Men

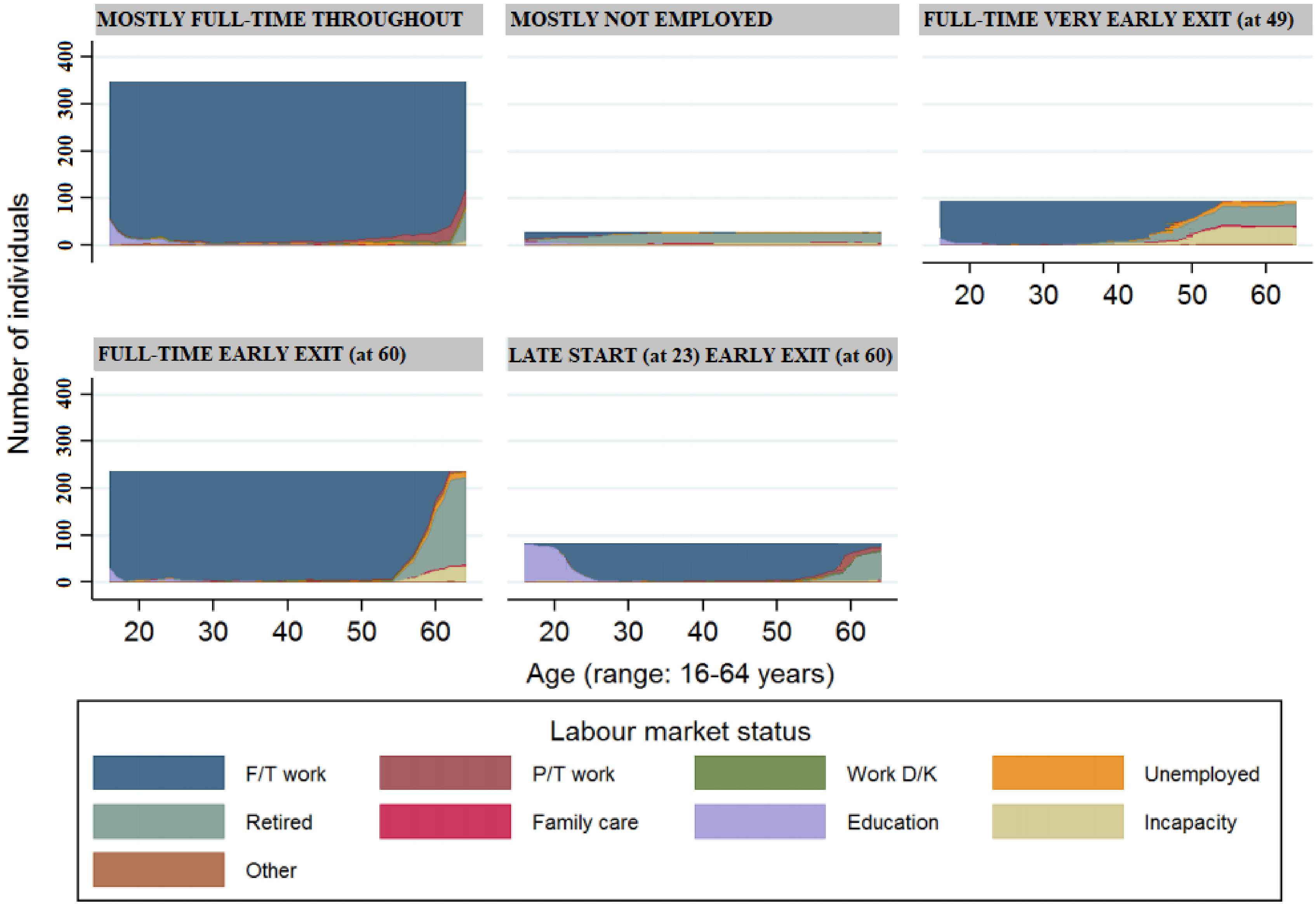

Supplementary Figure S2. Categories of employment histories – Women

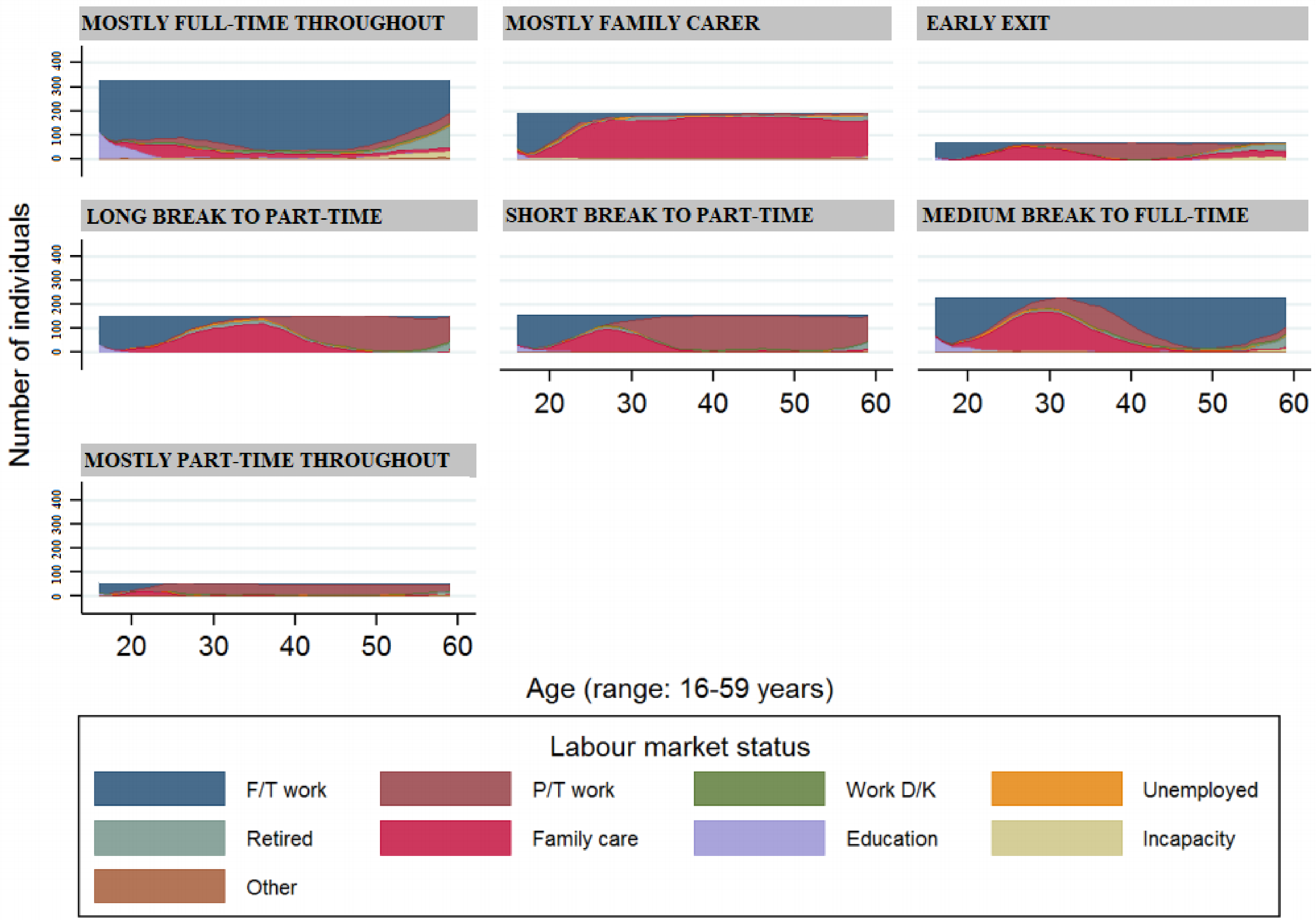

Supplementary Figure S3. Conditional response probabilities for each childhood variable in each of the four latent classes

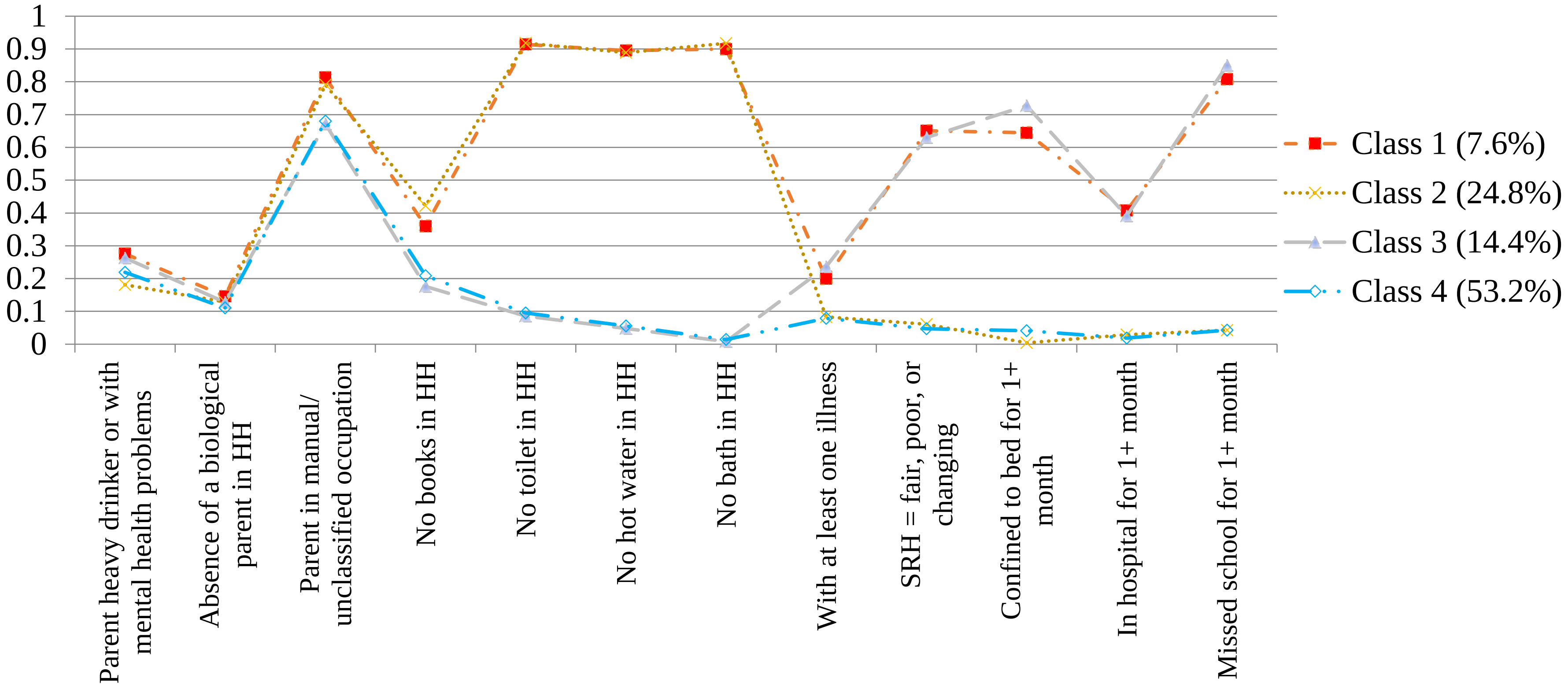

Source: ELSA Life History. Indicators included: whether one of the parents or guardians was a heavy drinker or had mental health problems; whether a biological parent was absent from the household; the main parental breadwinner's occupation (non-manual, manual, unclassified); the number of books in the household; the features of the accommodation (i.e. an inside toilet, fixed bath and a running hot water supply). Measures of health in childhood included: respondent's self-reported health in childhood; whether respondents had experienced any illnesses (such as polio, epilepsy or cancer); whether respondents had been confined to bed, stayed in the hospital, or missed school for more than one month.

The final number of latent classes chosen was based on several statistics (Pearson Chi-square, bootstrap likelihood ratio test and the Bayesian information criterion), as well as on substantive criteria and interpretability of the resulting categories. The entropy index was used to assess classification quality, with values approaching 1.0 indicating a favourable classification.

We labelled classes as 'Lower socio-economic circumstances and poor health' (Class 1), 'Lower socio-economic circumstances and good health' (Class 2); 'Higher socio-economic circumstances and poor health' (Class 3); and 'Higher socio-economic circumstances and good health' (Class 4). Note: we specified 2000 random sets of starting values, 50 optimizations and 50 iterations for the final stage, in order to ensure the replication of the best log likelihood and that solutions are not derived from local maxima. The entropy statistic obtained for such classification, i.e. a summary measure for the quality of classification, was equal to 0.89 indicating high classification accuracy. Also, the classification matrix indicated a high reliability of the classification. In our case, we obtained high class assignment probabilities for all classes (with values ranging from 0.93 for Class 1 to 0.95 for Classes 3 and 4).

Supplementary Table S2. Multilevel models of three measures of health by gender-specific categories of lifetime employment histories, beta coefficients and odd ratios (and 95% CIs) – results for MEN (N=794)

	Quality of Life			Somatic Health			Depressed (3+ CESD)		
	**Model I**	**Model II**	**Model III**	**Model I**	**Model II**	**Model III**	**Model I**	**Model II**	**Model III**
**Continuous work**	Ref.	Ref.	Ref.	Ref.	Ref.	Ref.	Ref.	Ref.	Ref.
**Weak attachment**	-1.894<br>(-3.54; -0.25)	-1.622<br>(-3.22; -0.20)	-1.789<br>(-3.36; -0.22)	-1.362<br>(-3.32; -0.40)	-1.074<br>(-2.01; -0.14)	-1.055<br>(-1.99; -0.13)	1.58<br>(0.70; 3.56)	1.11<br>(0.49; 2.48)	1.08<br>(0.49; 2.40)
**Exit at 60**	-0.718<br>(-2.01; 0.56)	-1.011<br>(-2.27; -0.24)	-0.781<br>(-2.01; 0.44)	-0.510<br>(-1.27; 0.25)	-0.472<br>(-1.21; 0.27)	-0.477<br>(-1.21; 0.26)	1.33<br>(0.68; 2.60)	1.22<br>(0.63; 2.36)	1.19<br>(0.62; 2.29)
**Late start, Exit at 60**	2.127<br>(0.28; 3.97)	0.681<br>(-1.17; 2.53)	0.663<br>(-1.14; 2.47)	*0.921*<br>(-0.07; 1.92	0.387<br>(-0.71; 1.48)	0.365<br>(-0.73; 1.46)	0.61<br>(0.21; 1.75)	0.99<br>(0.34; 2.87)	0.83<br>(0.28; 2.39)
**Age**	-0.208<br>(-0.39; -0.03)	-0.174<br>(-0.35; -0.00)	-0.174<br>(-0.34; -0.01)	-0.237<br>(-0.34; -0.13)	-0.205<br>(-0.30; -0.10)	-0.202<br>(-0.30; -0.10)	1.00<br>(0.92; 1.09)	0.98<br>(0.90; 1.07)	0.99<br>(0.91; 1.07)
**Wave**	-0.518<br>(-0.87; -0.17)	-0.618<br>(-0.97; -0.27)	-0.565<br>(-0.91; 0.21)	-0.353<br>(-0.53; -0.17)	-0.320<br>(-0.50; -0.14)	-0.319<br>(-0.50; -0.14)	0.90<br>(0.70; 1.16)	0.91<br>(0.85; 1.27)	0.88<br>(0.68; 1.14)
**Wave squared**	-0.065<br>(-0.13; 0.00)	-0.041<br>(-0.10; 0.02)	-0.038<br>(-0.10; 0.02)	*-0.027*<br>*(-0.06; 0.00)*	*-0.026*<br>*(-0.06; 0.01)*	*-0.025*<br>*(-0.06; 0.01)*	*1.05*<br>(1.00; 1.10)	1.05<br>(0.99; 1.09)	1.05<br>(1.00; 1.10)
**Wave*Labour histories**									
Weak attachment	0.247<br>(-0.11; 0.61)	0.262<br>(-0.10; 0.63)	0.289<br>(-0.08; 0.66)	0.204<br>(0.00; 0.41)	0.203<br>(0.01; 0.40)	0.197<br>(0.00; 0.39)	1.03<br>(0.84; 1.25)	1.04<br>(0.85; 1.27)	1.02<br>(0.84; 1.25)
Early exit	*0.227*<br>*(-0.01; 0.47)*	*0.256*<br>*(-0.03; 0.54)*	*0.253*<br>*(-0.03; 0.54)*	0.097<br>(-0.07; 0.26)	0.072<br>(-0.09; 0.23)	0.072<br>(-0.09; 0.23)	0.87<br>(0.73; 1.02)	0.89<br>(0.75; 1.05)	0.88<br>(0.74; 1.04)
Late start	0.435<br>(0.02; 0.85)	0.442<br>(0.03; 0.86)	0.415<br>(0.01; 0.83)	-0.016<br>(-0.21; 0.24)	-0.047<br>(-0.27; 0.18)	-0.036<br>(-0.26; 0.19)	0.95<br>(0.73; 1.25)	0.96<br>(0.73; 1.26)	1.00<br>(0.76; 1.31)
Never left employment because of ill health [a]	3.534<br>(2.18; 4.88)	3.07<br>(1.78; 4.37)	3.142<br>(1.88; 4.40)	3.437<br>(2.68; 4.19)	3.249<br>(2.52; 3.97)	3.212<br>(2.48; 3.94)	0.22<br>(0.13; 0.39)	0.26<br>(0.15; 0.46)	0.28<br>(0.16; 0.47)
1 o no periods of ill health in adulthood [b]	3.059<br>(1.57; 4.55)	2.78<br>(1.35; 4.22)	2.901<br>(1.51; 4.29)	3.361<br>(2.52; 4.19)	3.249<br>(2.44; 4.06)	3.222<br>(2.42; 4.03)	0.47<br>(0.25; 0.90)	0.53<br>(0.29; 0.97)	0.51<br>(0.28; 0.92)
Lower socio-economic circumstances, good health [c]	1.690<br>(-0.48; 3.86)	1.64<br>(-0.44; 3.73)	1.748<br>(-0.28; 3.78)	0.372<br>(-0.84; 1.59)	0.383<br>(-0.78; 1.55)	0.421<br>(-0.75; 1.59)	0.87<br>(0.34; 2.24)	0.96<br>(0.38; 2.40)	0.93<br>(0.38; 2.26)
Higher socio-economic circumstances, good health [c]	1.244<br>(-1.18; 3.67)	0.84<br>(-1.19; 2.87)	0.734<br>(-1.55; 3.02)	0.349<br>(-0.83; 1.53)	0.163<br>(-0.97; 1.31)	0.198<br>(-0.94; 1.34)	0.77<br>(0.31; 1.92)	1.37<br>(0.49; 3.87)	1.20<br>(0.43; 3.29)
Higher socio-economic circumstances, poor health [c]	1.239<br>(-0.86; 3.34)	0.650<br>(-1.69; 2.99)	0.987<br>(-0.98; 2.96)	0.096<br>(-1.26; 1.46)	-0.162<br>(-1.47; 1.16)	-0.124<br>(-1.45; 1.20)	1.02<br>(0.35; 2.95)	0.99<br>(0.41; 2.42)	0.90<br>(0.38; 2.14)

Intermediate [d]		0.467 (-0.82; 1.75)	0.603 (-0.65; 1.86)			0.495 (-0.23; 1.22)	0.562 (-0.16; 1.29)			0.66 (0.37; 1.18)	0.68 (0.38; 1.21)
Managerial and professional [d]		2.072 (0.89; 3.25)	2.041 (0.88; 3.20)			0.781 (0.11; 1.45)	0.804 (0.13; 1.47)			0.66 (0.38; 1.15)	0.79 (0.46; 1.36)
Own with mortgage [e]		0.515 (-1.09; 2.12)	0.601 (-0.97; 2.17)			0.561 (-0.29; 1.42)	0.626 (-0.23; 1.48)			0.47 (0.20; 1.10)	0.56 (0.24; 1.31)
Own outright [e]		1.466 (0.20; 2.73)	1.683 (0.44; 2.93)			0.719 (0.03; 1.40)	0.804 (0.11; 1.50)			0.41 (0.23; 0.72)	0.51 (0.29; 0.90)
Income (£10,000)		0.270 (0.04; 0.50)	0.279 (0.05; 0.50)			0.134 (0.01; 0.25)	0.134 (0.01; 0.25)			0.83 (0.69; 1.00)	0.84 (0.70; 1.00)
Wealth (£10,000)		0.002 (-0.00; 0.01)	0.002 (-0.00; 0.01)			0.003 (0.00; 0.01)	0.003 (0.00; 0.01)			1.00 (0.99; 1.00)	1.00 (0.99; 1.00)
Not a smoker [f]		0.264 (-0.80; 1.32)	0.263 (-0.78; 1.31).			0.041 (-0.52; 0.60)	0.040 (-0.52; 0.61)			0.54 (0.31; 0.92)	0.57 (0.33; 0.98)
Vigorous activity [g]		1.126 (0.64; 1.61)	1.130 (0.65; 1.61)			1.064 (0.81; 1.31)	1.040 (0.79; 1.29)			0.65 (0.45; 0.94)	0.64 (0.44; 0.92)
In a partnership [h]			-0.415 (-1.10; 0.27)				-0.464 (-0.84; -0.09)				0.64 (0.44; 0.92)
Never divorced/widowed [i]			1.777 (-0.48; 4.03)				1.598 (0.29; 2.90)				0.43 (0.17; 1.08)
No children [l]			0.573 (-0.98; 2.13)				0.259 (-0.64; 1.16)				1.62 (0.78; 3.34)
1 child [l]			-0.798 (-2.28; 0.69)				0.091 (-0.77; 0.95)				1.15 (0.58; 2.26)
2 children [l]			0.771 (-0.37; 1.92)				0.182 (-0.48; 0.84)				0.85 (0.49; 1.45)
Close relationships			0.114 (0.07; 0.15)				-0.000 (-0.02; 0.02)				0.92 (0.89; 0.96)
Contacts with family			0.028 (-0.07; 0.13)				-0.031 (-0.09; 0.03)				0.97 (0.91; 1.04)
Contacts with friends			0.490 (0.30; 0.68)				0.049 (-0.05; 0.15)				1.00 (0.88; 1.13)
Positive support by family			0.022 (-0.02; 0.06)				0.005 (-0.02; 0.03)				0.97 (0.95; 1.00)
Positive support by friends			0.054 (-0.02; 0.13)				0.018 (-0.02; 0.06)				0.98 (0.93; 1.03)
Constant	50.790 (37.8; 63.8)	46.414 (33.8; 58.9)	40.311 (27.9; 52.7)		11.274 (4.01; 18.6)	7.739 (0.68; 14.8)	5.83 (-1.39; 13.04)				

Sources: ELSA Waves 3-8 and ELSA Life History. Reference categories: a) Has left employment because of ill health; b) 2 or more periods of ill health in adulthood; c) In lower SEC and poor health in childhood; d) Routine and Manual social class; e) Non-owners; f) Smoker; g) No vigorous physical exercise; h) Not in a partnership; i) Has experience marital disruption (divorce of widowhood); l) 3 or more children. Own calculations.

Supplementary Table S3. Multilevel models of three measures of health by gender-specific categories of lifetime employment histories, beta coefficients and odd ratios (and 95% CIs) – results for WOMEN (N=1,140)

	Quality of Life			Somatic Health			Depressed (3+ CESD)		
	**Model I**	**Model II**	**Model III**	**Model I**	**Model II**	**Model III**	**Model I**	**Model II**	**Model III**
**Continuous work**	Ref.	Ref.	Ref.	Ref.	Ref.	Ref.	Ref.	Ref.	Ref.
**Weak attachment**	-0.802<br>(-2.09; 0.48)	-0.484<br>(-1.75; 0.77)	-0.621<br>(-1.89; 0.65)	-0.955<br>(-1.71; -0.20)	-0.649<br>(-1.29; -0.01)	-0.710<br>(-1.45; 0.03)	1.26<br>(0.77; 2.05)	1.08<br>(0.67; 1.75)	1.22<br>(0.75; 1.98)
**Long break**	0.696<br>(-0.88; 2.27)	0.726<br>(-0.81; 2.26)	0.689<br>(-0.85; 2.27)	0.522<br>(-0.39; 1.44)	0.604<br>(-0.28; 1.49)	0.583<br>(-0.32; 1.48)	0.62<br>(0.33; 1.17)	0.67<br>(0.36; 1.24)	0.77<br>(0.41; 1.43)
**Medium break**	1.400<br>(0.07; 2.73)	1.289<br>(0.01; 2.57)	1.329<br>(0.01; 2.64)	0.974<br>(0.19; 1.75)	0.931<br>(0.18; 1.68)	0.896<br>(0.12; 1.67)	0.68<br>(0.40; 1.16)	0.75<br>(0.45; 1.26)	0.81<br>(0.48; 1.38)
**Short break**	0.658<br>(-0.87; 2.19)	0.712<br>(-0.77; 2.20)	0.341<br>(-1.15; 1.84)	0.687<br>(-0.20; 1.76)	0.774<br>(-0.08; 1.63)	0.586<br>(-0.28; 1.46)	0.54<br>(0.28; 0.99)	0.52<br>(0.28; 0.96)	0.66<br>(0.35; 1.22)
**Age**	-0.150<br>(-0.29; -0.01)	-0.156<br>(-0.29; -0.01)	-0.132<br>(-0.26; -0.00)	-0.143<br>(-0.23; -0.06)	-0.139<br>(-0.22; -0.06)	-0.131<br>(-0.21; -0.05)	1.04<br>(0.99; 1.09)	1.04<br>(0.99; 1.09)	1.02<br>(0.97; 1.07)
**Wave**	-0.277<br>(-0.55; -0.01)	-0.337<br>(-0.62; 0.05)	-0.245<br>(-0.53; 0.03)	-0.094<br>(-0.25; 0.06)	-0.059<br>(-0.21; 0.09)	-0.042<br>(-0.20; 0.11)	1.11<br>(0.93; 1.32)	1.12<br>(0.93; 1.33)	1.08<br>(0.91; 1.29)
**Wave squared**	-0.018<br>(-0.06; 0.03)	-0.006<br>(-0.05; 0.04)	-0.016<br>(-0.06; 0.03)	-0.025<br>(-0.05; 0.00)	-0.027<br>(-0.05; -0.00)	-0.032<br>(-0.06; -0.01)	0.98<br>(0.95; 1.02)	0.99<br>(0.95; 1.02)	0.99<br>(0.96; 1.02)
**Wave*Labour histories**									
Weak attachment	0.067<br>(-0.18; 0.31)	0.116<br>(-0.14; 0.37)	0.123<br>(-0.13; 0.38)	0.057<br>(-0.09; 0.20)	0.032<br>(-0.10; 0.17)	0.038<br>(-0.10; 0.18)	0.93<br>(0.82; 1.05)	0.94<br>(0.82; 1.06)	0.93<br>(0.82; 1.06)
Long break	-0.028<br>(-0.34; 0.28)	-0.046<br>(-0.35; 0.26)	-0.048<br>(-0.35; 0.26)	-0.082<br>(-0.26; 0.10)	-0.093<br>(-0.26; 0.08)	-0.088<br>(-0.26; 0.08)	0.98<br>(0.83; 1.15)	0.98<br>(0.83; 1.15)	0.97<br>(0.83; 1.14)
Medium break	-0.036<br>(-0.30; 0.23)	-0.033<br>(-0.30; 0.23)	-0.047<br>(-0.31; 0.21)	-0.090<br>(-0.24; 0.06)	-0.112<br>(-0.26; 0.03)	-0.112<br>(-0.26; 0.04)	0.97<br>(0.82; 1.14)	0.92<br>(0.80; 1.06)	0.92<br>(0.80; 1.06)
Short break	0.068<br>(-0.24; 0.37)	0.096<br>(-0.20; 0.40)	0.084<br>(-0.22; 0.38)	-0.014<br>(-0.18; 0.15)	-0.007<br>(-0.17; 0.16)	-0.004<br>(-0.17; 0.16)	0.93<br>(0.81; 1.07)	0.96<br>(0.82; 1.13)	0.96<br>(0.82; 1.13)
Never left employment because of ill health [a]	3.127<br>(2.03; 4.23)	2.765<br>(1.70; 3.82)	2.628<br>(1.59; 3.66)	3.327<br>(2.70; 3.95)	3.169<br>(2.56; 3.77)	3.146<br>(2.54; 3.74)	0.40<br>(0.27; 0.57)	0.46<br>(0.33; 0.66)	0.46<br>(0.32; 0.65)
1 o no periods of ill health in adulthood [b]	3.317<br>(2.05; 4.57)	3.118<br>(1.90; 4.34)	3.221<br>(2.03; 4.41)	4.221<br>(3.50; 4.93)	4.074<br>(3.38; 4.76)	4.103<br>(3.41; 4.79)	0.31<br>(0.21; 0.47)	0.34<br>(0.23; 0.50)	0.35<br>(0.24; 0.52)
Lower socio-economic circumstances, good health [c]	1.899<br>(-0.08; 3.88)	1.950<br>(0.02; 3.88)	1.983<br>(0.10; 3.87)	0.913<br>(-0.21; 2.05)	0.896<br>(-0.20; 1.98)	0.958<br>(-0.12; 2.04)	0.61<br>(0.30; 1.20)	0.61<br>(0.31; 1.17)	0.64<br>(0.33; 1.21)
Higher socio-economic circumstances, good health [c]	0.585<br>(-1.28; 2.45)	0.464<br>(-1.35; 2.28)	0.260<br>(-1.73; 2.25)	0.633<br>(-0.43; 1.70)	0.512<br>(-0.51; 1.53)	0.547<br>(-0.47; 1.56)	0.91<br>(0.48; 1.72)	0.96<br>(0.52; 1.76)	0.89<br>(0.45; 1.74)
Higher socio-economic circumstances, poor health [c]	0.442<br>(-1.67; 2.55)	0.209<br>(-1.83; 2.54)	0.490<br>(-1.28; 2.26)	0.530<br>(-0.67; 1.73)	0.382<br>(-0.77; 1.53)	0.395<br>(-0.75; 1.54)	0.79<br>(0.38; 1.62)	0.86<br>(0.43; 1.71)	0.98<br>(0.54; 1.79)
Some education [d]	2.354<br>(1.36; 3.35)	1.229<br>(0.21; 2.24)	1.202<br>(0.21; 2.20)	1.333<br>(0.76; 1.90)	0.692<br>(0.11; 1.27)	0.666<br>(0.09; 1.24)	0.64<br>(0.45; 0.91)	0.96<br>(0.67; 1.35)	0.90<br>(0.64; 1.28)

Intermediate [e]		1.199 (0.16; 2.24)	1.160 (0.14; 2.17)			0.913 (0.32; 1.50)	0.885 (0.29; 1.47)			0.88 (0.62; 1.26)	0.86 (0.60; 1.22)
Managerial and professional[e]		1.819 (0.72; 2.92)	1.687 (0.60; 2.77)			1.197 (0.57; 1.82)	1.168 (0.55; 1.80)			0.66 (0.44; 0.97)	0.67 (0.45; 0.98)
Own with mortgage [f]		1.822 (0.16; 2.24)	1.572 (0.46; 2.68)			1.278 (0.65; 1.90)	1.144 (0.51; 1.77)			0.49 (0.30; 0.80)	0.74 (0.50; 1.09)
Own outright [f]		2.226 (1.28; 3.25)	1.942 (0.96; 2.92)			1.366 (0.82; 1.91)	1.174 (0.62; 1.73)			0.58 (0.39; 0.84)	0.58 (0.35; 0.95)
Income (£10,000)		0.154 (-0.01; 0.32)	0.145 (-0.03; 0.32)			0.033 (-0.06; 0.12)	0.025 (-0.06; 0.11)			0.79 (0.70; 0.89)	0.81 (0.72; 0.91)
Wealth (£10,000)		0.004 (-0.00; 0.01)	0.004 (-0.00; 0.01)			-0.001 (-0.00; 0.00)	-0.001 (-0.00; 0.00)			0.99 (0.99; 1.00)	1.00 (0.99; 1.00)
Not a smoker [g]		0.811 (-0.05; 1.67)	0.656 (-0.21; 1.52)			0.135 (-0.33; 0.60)	0.067 (-0.40; 0.54)			0.64 (0.43; 0.93)	0.71 (0.49; 1.04)
Vigorous exercise [h]		0.628 (0.23; 1.02)	0.639 (0.25; 1.03)			0.826 (0.61; 1.04)	0.827 (0.61; 1.04)			0.68 (0.54; 0.87)	0.68 (0.53; 0.86)
In a partnership [i]			0.782 (0.23; 1.33)				0.448 (0.14; 0.75)				0.48 (0.37; 0.63)
Never divorced/widowed [l]			0.978 (-0.23; 2.19)				0.381 (-0.32; 1.08)				0.98 (0.65; 1.48)
No children [m]			2.147 (0.70; 3.59)				0.617 (-0.21; 1.45)				0.71 (0.42; 1.19)
1 child [m]			0.600 (-0.62; 1.82)				0.555 (-0.15; 1.26)				0.62 (0.40; 0.96)
2 children [m]			1.462 (0.47; 2.45)				0.809 (0.24; 1.38)				0.80 (0.57; 1.13)
Close relationships			0.074 (0.04; 0.11)				0.016 (-0.00; 0.03)				0.97 (0.95; 0.99)
Contacts with family			0.096 (0.02; 0.17)				-0.005 (-0.05; 0.04)				0.93 (0.89; 0.97)
Contacts with friends			0.318 (0.17; 0.47)				0.041 (-0.04; 0.12)				0.91 (0.84; 0.99)
Positive support by family			0.033 (0.00; 0.06)				-0.003 (-0.02; 0.01)				0.98 (0.95; 0.99)
Positive support by friends			0.059 (0.00; 0.12)				-0.008 (-0.04; 0.02)				0.99 (0.97; 1.03)
Constant	44.567 (34.8; 54.3)	42.237 (32.8; 51.6)	35.059 (25.6; 44.5)		1.446 (-4.09; 6.98)	-0.328 (-5.66; 5.01)	-1.745 (-7.15; 3.66)				

Sources: ELSA Waves 3-8 and ELSA Life History. Reference categories: a) Has left employment because of ill health; b) 2 or more periods of ill health in adulthood; c) In lower SEC and poor health in childhood; d) No education; e) Routine and Manual social class; f) Non-owners; g) Smoker; h) No vigorous physical exercise; i) Not in a partnership; l) Has experience marital disruption (divorce of widowhood); m) 3 or more children. Own calculations

Supplementary Figure S4. Estimated trajectories of three health measures (Quality of Life, Somatic Health, and Depression) for men and women, by their gender-specific categories of lifetime employment histories.

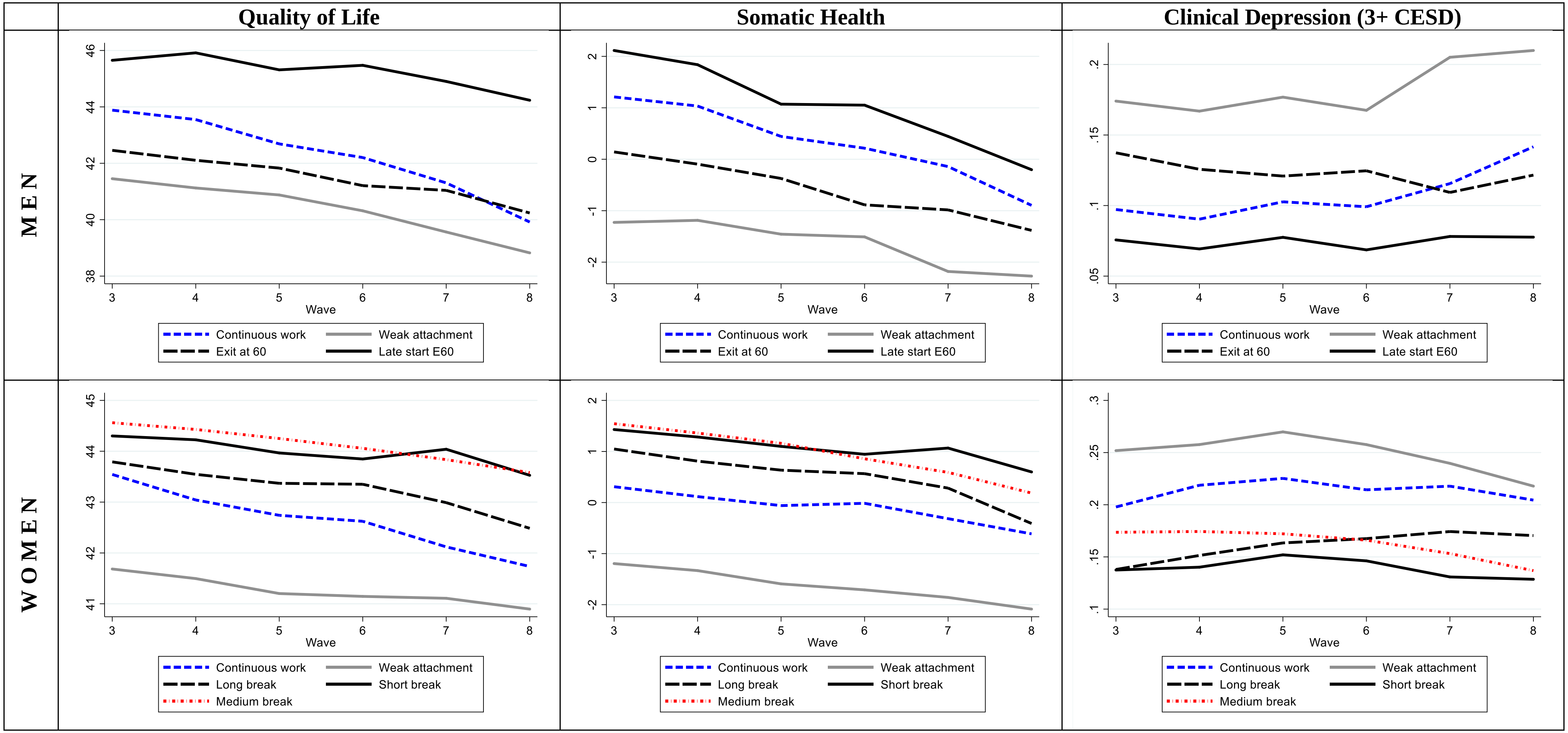

Sources: ELSA Waves 3-8 and ELSA Life History. Note: Curves represent mean trajectories by Wave for QoL and Somatic Health. For Depression, we present the estimated probability of reporting 3 or more CES-D symptoms.
